# Evaluating the effectiveness of prenatal exercise promotion strategies on the Xiaohongshu platform: Health beliefs, information quality, and source credibility

**DOI:** 10.1371/journal.pone.0330829

**Published:** 2025-09-02

**Authors:** Yan Fang, Di Wang

**Affiliations:** 1 Faculty of Humanities and Arts, Macau University of Science and Technology, Macau SAR, China; 2 Faculty of Arts, Hunan University of Information Technology, Changsha, Hunan, China; 3 Respiratory Disease AI Laboratory in Epidemic Intelligence and Applications of Medical Big Data Instruments, Faculty of Innovation Engineering, Macau University of Science and Technology, Macau SAR, China; Hamadan University of Medical Sciences, IRAN, ISLAMIC REPUBLIC OF

## Abstract

Although exercising during pregnancy offers numerous advantages, its prevalence in China remains relatively low. This is primarily attributed to the traditional Chinese belief that pregnancy is a period for rest and recuperation. To alter this perception, numerous individuals have promoted the benefits of prenatal exercise on Xiaohongshu, one of China’s most popular social media platforms. This study utilized the frameworks of the Health Belief Model (HBM) and the Heuristic - Systematic Model (HSM) to explore which strategies are effective in these promotional efforts. A total of 5,016 posts promoting prenatal exercise were identified. From these, 500 samples were randomly selected for coding. Negative binomial regression analysis was conducted to assess the influence of the constructs of HBM and HSM on public engagement. The Kruskal-Wallis test was used to compare various information sources’ differential effects. The results indicated that emphasizing the benefits, self-efficacy, and barriers to exercise significantly impact audience engagement in the context of social media information regarding exercise during pregnancy. Healthcare professionals and pregnant and postpartum women are the most influential information sources in attracting audience engagement. Moreover, source credibility significantly impacts public engagement, and information completeness positively increases the likelihood of favorites. These findings are valuable for optimizing the design of pregnancy exercise promotion information on social media, obtaining social support for prenatal exercise, and contributing to women’s health and well-being.

## 1 Introduction

Numerous studies have demonstrated the substantial benefits of moderate exercise during pregnancy for both the expectant mother and the fetus [1–3]. Moreover, the World Health Organization and many countries have issued guidelines about exercise during pregnancy [4]. These guidelines strongly advocate that all pregnant women without contraindications should exercise regularly throughout their pregnancy. However, a relatively small proportion of women partake in sufficient exercise during pregnancy to derive optimal health benefits [5–9]. This phenomenon is particularly pronounced in China. Traditional Chinese concepts of pregnancy and childbearing uphold that remaining stationary during pregnancy is more favorable than engaging in exercise [10–11]. As a result, women not only lack a structured exercise regimen during pregnancy but also significantly curtail their daily activities. This is substantiated by a longitudinal study that indicates that the exercise level of pregnant and postpartum Chinese women during pregnancy is relatively low [12]. For instance, in Shanghai, a mere 2.8% of women exercised adequately during pregnancy [9].

Meanwhile, traditional Chinese beliefs about pregnancy place extraordinary emphasis on nutrition and calorie intake. This overemphasis significantly elevates the risk of obesity, gestational diabetes mellitus, gestational hypertension, macrosomia, and other pregnancy-related diseases, all of which pose serious threats to the health of both the mother and the infant [13]. Additionally, it may have a long-term impact on the mental health of pregnant women.

In today’s digital era, social media has emerged as a crucial avenue for expectant mothers to access information regarding motherhood [14]. However, prior research on exercise during pregnancy has predominantly been concentrated within the medical domain. It generally focused on areas such as pregnant women’s attitudes towards exercise during pregnancy in offline contexts [15], the safety and efficacy of exercise during pregnancy, and the factors that act as barriers to exercise during pregnancy [16–20]. To date, relatively few studies have delved deeply into exploring the online promotion strategies of prenatal exercise. Thus, this study aims to fill this gap by exploring the effect of promotion strategies of prenatal exercise on Xiaohongshu, a highly vibrant social media platform among young women in China, with over 96% of users selecting it as the daily primary channel for learning about mother-and-baby information.

Applying the Health Belief Model (HBM) and Heuristic-Systematic Model (HSM) frameworks, this study examines promotion strategies embedded in pregnancy exercise messages on social media and their influence on public engagement. Moreover, it is intended to contribute to optimizing the social media design of pregnancy exercise promotion messages, thereby facilitating the accurate dissemination and efficient utilization of such messages.

## 2 Literature review

### 2.1 Health belief model

The Health Belief Model (HBM) is an important framework in health communication research. It is designed to explain, predict, and influence health-related behaviors [21,22]. Initially put forward by Hochbaum, Rosenstock, and Kegels in the 1950s, the HBM aimed to account for why individuals refrain from adopting preventive health behaviors [23].

After decades of development, the model now includes six factors: perceived susceptibility, severity, benefits, barriers, self-efficacy, and cues to action. Perceived severity pertains to an individual’s perception of the extent of harm stemming from a health issue. In contrast, perceived susceptibility refers to an individual’s assessment of the probability of developing a specific health problem. According to the HBM, the greater an individual’s sense of vulnerability to specific health problems and the more severe they perceive the consequences of inaction to be, the higher the likelihood they will take steps to avert the health problem [24]. Perceived benefits refer to an individual’s judgment of the benefits of adopting a behavior. Research has shown that knowledge about the benefits of exercise during pregnancy will lead to more positive attitudes toward exercise [25–27]. Perceived barriers are individuals’ judgments about the barriers they may face in adopting a behavior [28]. Perceived self-efficacy refers to an individual’s perception of his or her ability to successfully perform the suggested behavior [29]. Cues to action refer to external stimuli or prompts that motivate individuals to adopt preventive health behaviors [30].

Previous studies have established a positive correlation between the HBM constructs (except barriers) and the level of exercise among pregnant women during pregnancy with a cross-sectional survey [31]. To what extent the HBM constructs help design promotion messages on prenatal exercise, particularly in online environments, remains to be examined. Since the focus of this study was the analysis of social media posts related to the promotion of exercise during pregnancy, and nearly all the posts in our sample had elements prompting action, we did not incorporate the construct of “cues to action” in the study. Based on this, we raise the following hypotheses:

H1. Compared to other posts, posts that emphasize the severity of the negative effects of not exercising during pregnancy will attract more (a.) likes and (b.) favorites.

H2. Compared to other posts, posts that emphasize the susceptibility to the negative effects of not exercising during pregnancy will attract more (a.) likes and (b.) favorites.

H3. Compared to other posts, posts that emphasize the benefits of exercise during pregnancy will attract more (a.) likes and (b.) favorites.

H4. Compared to other posts, posts that emphasize barriers to exercise during pregnancy will attract fewer (a.) likes and (b.) favorites.

H5. Compared to other posts, posts that include prompts to boost self-efficacy in exercising during pregnancy will attract more (a.) likes and (b.) favorites.

### 2.2 Heuristic-systematic model

In addition to the factors of health beliefs, the mode of information processing will also impact the audiences’ attitudes and behavior [32]. The Heuristic-Systematic Model (HSM) is an information processing theory proposed by Chaiken in 1980 to explain how individuals make judgments and decisions when confronted with persuasive messages. The model posits that people could use systematic and heuristic information-processing strategies to process information [33]. Systematic processing demands more cognitive resources and entails in-depth information analysis and logical reasoning. Accepting a persuasive message from a systematic route can arise from people carefully examining and evaluating the content of the information.

In contrast, heuristic processing depends on rapid, automated judgments. Accepting a persuasive message from a heuristic route can occur based on simple cues, such as emotions, stereotypes, or straightforward cues that do not necessitate excessive cognitive effort [34,35]. In the research on social media information dissemination mechanisms, HSM has been employed to analyze the impacts of heuristic and systematic cues on the effectiveness of information dissemination. Empirical evidence has demonstrated that the combination of heuristic cues and systematic cues can effectively enhance the public’s acceptance of health information, thereby facilitating the adoption of health-promoting behaviors [36].

Prior research has shown that information quality serves as the most important systematic cue. High-quality information typically denotes reliability and increases the message’s persuasiveness [37,38]. Information quality is usually measured by accuracy and completeness [39,40]. Information accuracy refers to the degree of correctness and clarity of the information.Providing accurate health information is crucial for maintaining the credibility of information sources. Morever, the accuracy of information will affect the correctness of individual decisions [40,41]. Information completeness refers to the extent to which health information is comprehensive and sufficient in covering a health topic. It is an important criterion for individuals when making decisions [42,43]. As the completeness of information increases, the audience’s trust in the information is likely to rise [44]. Conversely, incomplete health information may mislead the audience into making ill-informed decisions [42,44].

Source credibility has been found to be one of the most prevalent heuristic cues. When individuals perceive that posts on social media originate from individuals or organizations with high credibility, they tend to affirm the value of these posts [45]. The greater the credibility of the sources, the more persuasive these sources are considered to be [46]. Therefore, we predict these sources will also attract more public engagement on social media.

Based on the above findings, the following hypothesis is proposed:

H6. Compared to other posts, posts with high information completeness will attract more (a.) likes and (b.) favorites.

H7. Compared to other posts, posts with high information accuracy will attract more (a.) likes and (b.) favorites.

H8. Compared to other posts, posts from credible sources will attract more (a.) likes and (b.) favorites.

**Fig 1:**
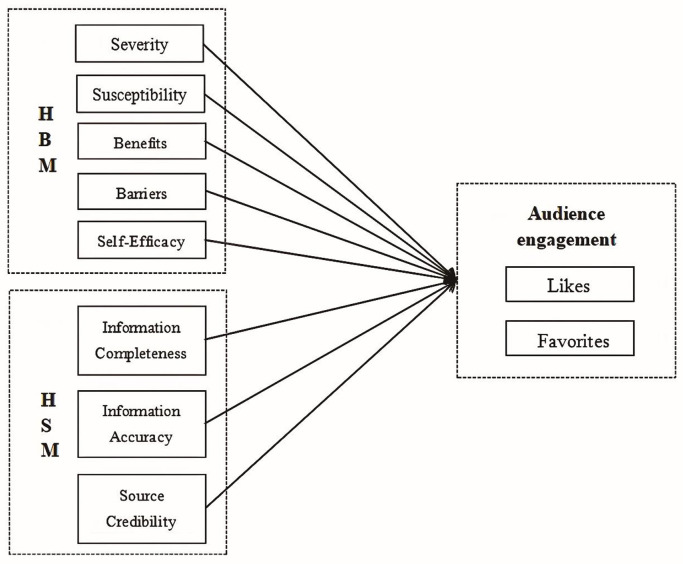
Conceptual framework of the study.

The framework of the study is shown in Fig 1.

### 2.3 Information sources and their application of HBM and HSM constructs

Individuals prefer information from highly trusted sources to acquire valuable information with minimal risk [47]. Regarding prenatal exercise, healthcare professionals, including midwives, are often regarded as the most dependable sources of information [13,48].

In addition to healthcare professionals, fitness professionals possess specialized knowledge in exercise instruction during pregnancy. Research suggests that sports professionals play an important role in providing information on exercise options and techniques for prenatal exercise [27]. However, due to its inherent commercial attributes, its credibility may be lower than that of healthcare professionals.

Furthermore, some pregnant women seek social support from pregnant and postpartum social media influencers [49]. Their emotional connection, shared experiences, and trust inpregnant and postpartum social media influencers make them more receptive to their advice [50,51].

Besides the sources mentioned above, information sources also encompass commercial entities such as fitness centers, maternity centers, for-profit healthcare providers, and ordinary individuals. Given that different information sources may yield varying persuasive effects, the following research questions are raised:

RQ 1. Which information sources of prenatal exercise attract the most likes and favorites on social media?

RQ 2. Do information sources of prenatal exercise vary in their use of the HBM and HSM constructs?

## 3 Method

### 3.1 Sampling

We used Python to download all the data on the Xiaohongshu platform from November 27, 2016 (the first post on the topic) to December 31, 2023(when the study was conducted). The primary keywords were words related to pregnant women, and the secondary keywords covered various expressions related to exercise and the most common types of pregnancy-related exercises. Specifically, the primary keywords included "pregnancy period, gestation period, pregnancy, and pregnant women"; the secondary keywords included "exercise, workout, fitness, sports activity, aerobic, walking, jogging, swimming, resistance training, muscle training, strength training, resistance band, dumbbell, pelvic floor muscle training, Kegel, stretching, yoga, and Pilates". All keywords were searched in Chinese.

After removing duplicates, 9,618 posts were obtained. Subsequently, all data underwent manual screening, with irrelevant posts excluded, resulting in a final dataset of 5,016 posts. We employed the DiVoMiner® online platform to conduct system-driven automated random sampling, selecting 500 posts (≈10%) to establish the coding framework, thereby effectively mitigating human bias. Additionally, we further compared key structural indicators at the account level between the sample and the total dataset to verify its representativeness. 

Specifically, the 500 samples involved 359 accounts, accounting for 10.42% of the total 3,445 accounts. This proportion is similar to the sample size proportion, 9.97% (500 out of 5016 posts). The mean number of posts published by sample accounts is 1.39, whereas the corresponding figure for the total account population is 1.46. The disparity in the average posts per account is minimal, characterized by an absolute difference of 0.07 and a relative difference of 4.79%.

The consistency of the aforementioned structural indicators suggests that the 500 samples acquired via random sampling exhibit a high degree of similarity to the total dataset with respect to the distribution characteristics of information source accounts.

All data were publicly accessible on the Xiaohongshu Platform, and the data collection and analysis methods were conducted in compliance with the terms and conditions of the Xiaohongshu Platform.

### 3.2 Operationalization of variables

#### 3.2.1 Dependent variables: The number of likes and favorites.

Drawing on previous research and taking into account the characteristics of the Xiaohongshu platform, the number of likes and favorites were selected as representatives of public engagement. The number of likes has been widely used as a crucial indicator of audience engagement, which, to some extent, reflects the audience’s preference and approval of the post [36]. Favorites signify that the content is useful and has a certain value. By hitting the favorites button, the post is bookmarked for future browsing, use, or sharing, which, to some extent, reflects the audience’s approval of the content [52]. Thus, the number of likes and favorites is used to evaluate the effectiveness of promotion strategies.

The reason we did not include comments and shares in the public engagement analysis was based on the following considerations. Firstly, the number of comments alone cannot reflect audience attitudes, as comments may be positive, negative, or neutral. Secondly, the specific quantity of shares is not publicly available on the Xiaohongshu platform. Consequently, comments and shares were not included in the study.

#### 3.2.2 Independent variables.

The independent variables consisted of constructs related to the HBM and HSM models. The HBM constructs include severity, susceptibility, benefits, barriers, and self-efficacy, and the HSM model includes systematic cues and heuristic cues. Systematic cues consist of information completeness and accuracy, while heuristic cues only include the credibility of the source. The operationalization definitions of the variables are shown in Table 1.

**Table 1:** Definitions and examples of variable operationalization.

	Variable	Operational Definition	Example
**HBM**	Severity	Specify the potential adverse reactions or issues that may arise during pregnancy.	Example 1: “Many expectant mothers fail to control their weight during pregnancy, resulting in the fetus being too large and experiencing severe bleeding, tearing, and other complications during delivery.”
			Example 2: “Pelvic floor muscles will stretch excessively due to pregnancy and childbirth, which will easily lead to pelvic floor muscle relaxation, organ prolapse, urinary incontinence and urinary retention, etc. Regular pelvic floor muscle training can effectively alleviate the problem.”
	Susceptibility	Specify the likelihood and probability of adverse reactions or issues during pregnancy.	Example 1: “Do you know? Nearly half of all women in our country experience different degrees and forms of pelvic problems after childbirth, such as stress incontinence, pelvic organ prolapse, and other pelvic floor dysfunction disorders (PFD).”
			Example 2: “It is estimated that up to 43% of pregnant women become overweight during pregnancy. Can exercise during pregnancy control weight? Is it safe? Find it out today.”
	Benefits	State the benefits of prenatal exercise, which include benefits for maternal physical health, benefits for maternal mental health, benefits for maternal appearance, benefits for labor and delivery, and benefits for the fetus.	Benefits for maternal physical health refer to physiological improvements, such as enhanced cardiovascular fitness and reduced risk of gestational diabetes.
			Benefits for maternal mental health refer to positive psychological effects, including reduced stress and improved mood.
			Benefits for maternal appearance refer to improvements in body image, such as maintaining a toned physique and improving body posture.
			Benefits for labor and delivery refer to childbirth facilitation, including shorter labor duration and reduced risk of perineal tears.
			Benefits for the fetus refer to positive developmental outcomes, such as improved fetal growth and lower risk of macrosomia.
	Barriers	In the post, state the barriers to prenatal exercise, including potential risk, cognitive, conditional, and difficulty-related barriers.	Risk barriers refer to perceived or actual threats to maternal or fetal health that may arise from prenatal exercise.
			Cognitive barriers are beliefs contradicting evidence-based guidelines for regular, moderate-intensity exercise during pregnancy, such as the misconception that exercise is unsafe in the first trimester.
			Conditional barriers refer to external factors that may hinder prenatal exercise participation, such as the need for specialized equipment, professional instructors, or prenatal exercise programs, all of which can incur additional economic costs.
			Difficulty-related barriers refer to challenges associated with the complexity of implementing preventive exercise measures, particularly in the context of prenatal fitness.
	Self-Efficacy	Encouraging statements that suggest exercise is easy, which can stimulate and enhance the audience's confidence in exercising during pregnancy.	Example 1: “This exercise is beginner-friendly, even for sisters with no prior exercise background. You can pick it up quickly. It’s simple, uncomplicated, and won’t leave you flustered. Before you know it, you can control your weight!”
			Example 2: “Come on, sisters! Stick with it for a week, and you’ll find that exercise isn’ t as difficult as you thought. Even someone like me, who would rather lie down than sit, has managed to keep it up. You can do it too!”
	Information Completeness	The information completeness of a post is evaluated based on key recommendations from the WHO and Chinese experts [53,54]. Specifically, suppose a post covers three or more of the five key points (exercise benefits, types of exercise, total amount of exercise, safety tips, and risk precautions). In that case, it is judged to be highly complete. On the contrary, if fewer than three information points were included, it is considered low integrity.	Example 1: “Regular exercise during pregnancy is safe and can also help us with vaginal delivery and prevent side-cutting tears! Appropriate strength training can increase our muscle strength and stabilize the pelvis, but if pregnant women experience discomfort during exercise, they should stop immediately.” This statement integrates four critical components: safety assurance, health benefits, exercise modalities, and risk precautions, thus coded as high completeness (score = 4).
			Example 2: “To achieve healthy fetal growth without excessive maternal weight gain, prenatal exercise is essential. Options include home-based aerobic routines or supervised clinical Pilates sessions to improve muscular elasticity. Overall, maintaining exercise is strongly recommended.” This statement contains only two elements: health benefits and exercise modalities, coded as low completeness (score = 2).
**HSM**	Information Accuracy	The accuracy of the information description in the post is judged as high if it is consistent with the WHO’s guidelines and expert recommendations [53,54], and low if it contradicts.	Example 1: “During the first trimester of pregnancy, exercise should be avoided as much as possible. During the second trimester, moderate exercise can be carried out, but the duration should not be too long, about 3 - 4 times a week, 20 - 30 minutes each time.” This statement contradicts the WHO’s recommendation, which states that pregnant women without exercise contraindications should carry out regular exercise throughout pregnancy, with no less than 150 minutes of moderate-intensity exercise per week. Therefore, it is coded as low accuracy.
			Example 2: “In fact, the risks of exercise during pregnancy are very low. For the health of both yourself and the baby, it is recommended that expectant mothers without contraindications for pregnancy-related exercise can carry out moderate-intensity exercise throughout pregnancy. You can choose your favorite exercise method, but remember to exercise for no less than 150 minutes weekly.” This statement aligns with the WHO’s guidelines and was coded with high accuracy.
	Source Credibility	The credibility of the post publisher is comprehensively judged based on the publisher’s account information and homepage profile. Healthcare professionals or pregnant and postpartum women are considered high-credibility sources; fitness professionals; ordinary individuals, and commercial entities are classified as low-credibility sources.	Example 1: For instance, a publisher’s homepage information states that the person is a chief physician in the Department of Obstetrics and Gynecology at Shanghai First Maternity and Infant Health Hospital. Since the publisher is a healthcare professional, the source is coded as high credibility.
			Example 2: For example, a publisher’s homepage information describes it as a pregnancy education center specializing in pregnancy exercise and postpartum recovery. As this publisher is a commercial entities, it is coded as a low-credibility source.

#### 3.2.3 Control variable.

Given that the number of followers of a publisher may influence public engagement, it was included as a control variable in the evaluation.

#### 3.2.4 Inter-coder reliability.

Two graduate students carried out the data coding. Prior to the official initiation of the study, the researchers engaged in multiple training activities and communication sessions with the coders. Subsequently, 10% of the samples were randomly selected to test inter-coder reliability. For the discrepancies that emerged, the differences were gradually minimized through in-depth discussions and negotiations, and a consensus was eventually achieved. For example, during the coding process, two coders disagreed regarding the sentence: “Pregnant mothers will inevitably experience various discomforts as they experience significant changes in hormone secretion. As our ligaments become lax, the joints will be easily sprained.” One coder contended that the sentence did not convey susceptibility because it did not explicitly state the probability of an adverse reaction. However, the other coder noted that although degree words such as inevitably and easily did not present numerical probability values, they did imply a certain degree of susceptibility. The researcher and the coders engaged in an in-depth discussion on this issue. Given that social media posts are typically created by non-experts and rarely contain precise probability statements, a consensus was reached about this type of statement, which expressed a high probability of an adverse condition and met the coding criteria for susceptibility. Cohen’s kappa coefficient ranges from 0.85 to 1.00, reflecting good inter-coder reliability.

## 4 Results

### 4.1 Application of HBM and HSM constructs in promoting exercise during pregnancy

All HBM framework constructs were represented in the posts, with benefits appearing most frequently (N = 460, 92%), followed by barriers (N = 304, 60.8%) and self-efficacy (N = 225, 45%), and severity (N = 96, 19.2%) and susceptibility (N = 51, 10.2%) appearing less frequently. Second, concerning the HSM constructs, for systematic cues, most messages were high in information completeness (N = 368, 73.6%), but the accuracy of the messages was poor (N = 127, 25.4%). For heuristic cues such as source credibility, most posts were from high-credibility sources (N = 388, 77.6%), with about a fifth from low-credibility sources (N = 112, 22.4%).

### 4.2 Impact of HBM and HSM framework application on audience engagement

As our dependent variables (likes and favorites) are overdispersed count data, we use negative binomial regression to do the analysis, as linear regression is not the most appropriate for count data [55]. Negative binomial regression is similar to regular linear regression, except that the dependent variable is a count of observations that follows a negative binomial distribution; that is, the possible values of the dependent variable are non-negative integers, such as 0, 1, 2, 3, etc. Therefore, we chose negative binomial regression to examine the impact of HBM and HSM constructs on user engagement. Considering that the number of followers of the information source may affect public engagement, it is included as a control variable.

As shown in Table 2, in terms of likes, two HBM constructs positively predicted the number of likes (benefits, B = 1.119, *p* = 0.001; self-efficacy, B = 0.446, *p* = 0.024), while one variable negatively predicted the number of likes (barriers, B = −0.803, *p* = 0.003). Thus, hypotheses H3a, H4a, and H5a were supported. Similarly, in terms of favorites, the same two HBM constructs positively predicted the number of likes (benefits, B = 0.823, *p = *0.009; self-efficacy, B = 0.478, *p = *0.018), while one variable negatively predicted the number of likes (barriers, B = −0.567, *p = *0.018). Thus, hypotheses H3b, H4b, and H5b were also supported. However, the effect of severity and susceptibility on audience engagement was insignificant, so hypotheses H1a, H1b, H2a, and H2b were not supported.

**Table 2:** Predictors of likes and favorites using a negative binomial regression model.

Variables	Likes				Favorites			
	B	Exp(B)	Wald χ²	P value	B	Exp(B)	Wald χ²	P value
Severity	−0.287	0.751	1.096	0.295	−0.032	0.969	0.14	0.907
Susceptibility	0.635	1.887	1.436	0.231	0.302	1.353	0.430	0.512
Benefits	1.119	3.063	10.415	0.001	0.823	2.277	6.852	0.009
Barriers	−0.803	0.448	9.054	0.003	−0.567	0.567	5.603	0.018
Self-Efficacy	0.446	1.562	5.063	0.024	0.478	1.613	5.564	0.018
Information Completeness	0.324	1.383	0.945	0.331	0.707	2.027	7.784	0.005
Information Accuracy	0.042	1.043	0.020	0.886	−0.397	0.672	2.245	0.134
Source Credibility	0.820	2.270	6.100	0.014	1.335	3.800	25.793	<0.001
Followers	9.061E-6	1.000	19.582	<0.001	8.278E-6	1.000	20.511	<0.001

Regarding HSM constructs, heuristic cue source credibility had a significant positive effect on the number of likes, whereas a high credibility source (B = 0.820, *p = *0.014) gained more likes. In contrast, none of the systematic cues had a significant effect. Therefore, hypothesis H8a was supported, while H6a and H7a were not supported. In addition, systematic cue information completeness (B = 0.707, *p = *0.005), as well as heuristic cue source credibility (B = 1.335, **p* *< 0.001), significantly increased the likelihood of favorites. High completeness information and high credibility sources gained more favorites, but there was no significant effect on information accuracy. Therefore, hypotheses H6b and H8b were supported, while H7b was not.

Next, we examined the specific benefits and barriers to exercise during pregnancy. The frequency of the specific benefits mentioned, from high to low, was as follows: the benefits to the physical health of pregnant women (N = 370, 74%), the benefits to the appearance of pregnant women (N = 310, 62%), the benefits to labor and delivery (N = 244, 48.8%), the benefits to the fetus (N = 222, 44.4%), and the benefits to the mental health of pregnant women (N = 107, 21.4%).

Potential risk barriers (N = 241, 48.2%) and cognitive barriers (N = 161, 32.2%) were mentioned frequently, while conditional barriers (N = 64, 12.8%) and difficulty-related barriers (N = 15, 3%) were mentioned less frequently.

We used negative binomial regressions to further test the relationship between specific types of benefits and barriers and public engagement (Table 3). Among the subthemes related to benefits, emphasizing the benefits of appearance (Likes: B = 0.716, *p* = 0.021; Favorites: B = 0.696, *p = *0.026) and the benefits of labor and delivery (Likes: B = 0.422, **p* *= 0.043; Favorites: B = 0.444, *p = *0.039) were likely to receive more likes and favorites compared to posts that did not mention such benefits. In contrast, emphasizing the benefits to the fetus was likely to receive fewer likes and favorites (Likes: B = −0.599, *p = *0.030; Favorites: B = −0.568, *p = *0.043).

**Table 3:** Relationship between benefit and barrier subthemes and public engagement.

	Variables	Frequency (n, %)	Likes				Favorites			
			B	Exp(B)	Wald χ²	p-value	B	Exp(B)	Wald χ²	p-value
**Benefits**	The benefits to the physical health of pregnant women	370 (74%)	−0.011	0.989	0.001	0.971	−0.008	0.992	0.001	0.977
	The benefits to the mental health of pregnant women	107 (21.4%)	−0.052	0.949	0.059	0.808	−0.186	0.830	0.711	0.399
	The benefits to the appearance of pregnant women	310 (62%)	0.716	2.046	5.294	0.021	0.696	2.006	4.971	0.026
	The benefits of labor and delivery	244 (48.8%)	0.422	1.525	4.101	0.043	0.444	1.560	4.250	0.039
	The benefits to the fetus	222 (44.4%)	−0.599	0.549	4.737	0.030	−0.568	0.566	4.085	0.043
**Barriers**	Potential risk barriers	241 (48.2%)	−0.209	0.811	0.611	0.434	−0.130	0.878	0.239	0.625
	Cognitive barriers	161 (32.2%)	−0.619	0.538	8.157	0.004	−0.522	0.594	6.019	0.014
	Conditional barriers	64 (12.8%)	−0.770	0.463	5.594	0.018	−1.033	0.356	8.167	0.004
	Difficulty-related barriers	15 (3%)	1.430	4.179	7.870	0.005	1.876	6.529	12.247	<0.001
	Followers		1.205E-5	1.000	28.650	<0.001	1.215E-5	1.000	27.806	<0.001

Among the subthemes regarding barriers, emphasizing cognitive barriers (Likes: B = −0.619, *p = *0.004; Favorites: B = −0.522, *p = *0.014) and conditional barriers (Likes: B = −0.770, *p = *0.018; Favorites: B = −1.033, *p = *0.004) received fewer likes and favorites compared to posts that did not mention such barriers. In contrast, emphasizing difficulty-related barriers received few likes and favorites (Likes: B = 1.430, *p = *0.005; Favorites: B = 1.876, **p* *< 0.001).

### 4.3 Information sources and public engagement

As shown in Table 4, pregnant and postpartum women were the most prominent group of publishers (N = 346, 69.2%), followed by commercial entities (N = 54, 10.8%), healthcare professionals (N = 42, 8.4%), fitness professionals (N = 39, 7.8%), and ordinary individuals (N = 19, 3.8%).

**Table 4:** Kruskal-Wallis test results are used to find information sources and public engagement.

		Healthcare Professionals (N = 42)	Pregnant and Postpartum Women (N = 346)	Fitness Professionals (N = 39)	Ordinary Individuals (N = 19)	Commercial Entities (N = 54)	Kruskal-Wallis Test Statistics	*p*
**Likes**	Mean ranks	307.98	273.59	183.13	100.97	159.10	65.930	<0.001
	Medians	352	179	35	8	24.5		
**Favorites**	Mean ranks	314.96	273.18	174.49	95.24	164.55	68.754	<0.001
	Medians	511	221	25	5	28.5		

Due to the non-normal distribution of the number of likes and favorites, the Kruskal-Wallis nonparametric tests were employed to explore the relationship between information sources and public engagement. The Kruskal-Wallis test is capable of determining whether a statistically significant difference exists in the median values across distinct information sources, with its analytical basis rooted in the discrepancies in average ranks among groups.

The results showed a significant positive effect of information sources on both the number of likes (H = 65.930, *p* < 0.001) and the number of favorites (H = 68.754, *p* < 0.001). Public engagement demonstrated the highest levels when healthcare professionals served as the information source (likes: mean rank = 307.98, median = 352; favorites: mean rank = 314.96, median = 511), followed by posts from pregnant and postpartum women (likes: mean rank = 273.59, median = 179; favorites: mean rank = 273.18, median = 221). Other sources attracted significantly lower engagement. From the post hoc multiple comparisons (Table 5), posts made by healthcare professionals and pregnant and postpartum women significantly outperformed those made by fitness professionals, commercial entities, and ordinary individuals in terms of the number of likes and favorites.

**Table 5:** Post-hoc comparison results of Kruskal-Wallis test for information source and audience engagement.

	Likes		Favorites	
	Z-score	*p*	Z- score	*p*
Ordinary Individuals – Commercial Entities	−58.128	1.000	−69.309	0.721
Ordinary Individuals – Fitness Professionals	82.155	0.421	79.250	0.499
Ordinary Individuals – Pregnant and Postpartum Women	172.619	<0.001	177.947	<0.001
Ordinary Individuals – Healthcare Professionals	207.003	<0.001	219.727	<0.001
Commercial Entities – Fitness Professionals	24.026	1.000	9.941	1.000
Commercial Entities – Pregnant and Postpartum Women	114.491	<0.001	108.637	<0.001
Commercial Entities – Healthcare Professionals	148.874	<0.001	150.418	<0.001
Fitness Professionals – Pregnant and Postpartum Women	90.464	0.002	98.696	0.001
Fitness Professionals – Healthcare Professionals	124.848	0.001	140.477	<0.001
Pregnant and Postpartum Women – Healthcare Professionals	34.384	1.000	41.781	0.767

### 4.4 Application of HBM and HSM aconstructs across diverse information sources

**Fig 2:**
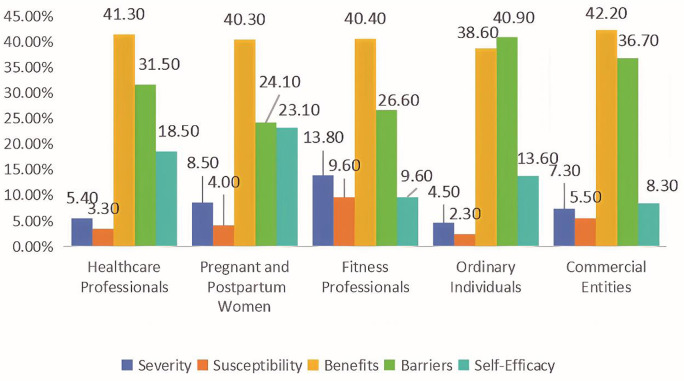
Use of HBM constructs across different information sources.

**Fig 3:**
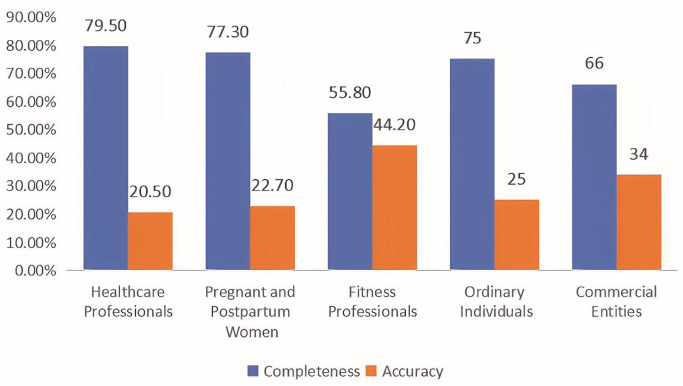
Informational quality of posts by different information source types.

As depicted in Figs 2 and 3, various information sources represent the constructs of HBM and HSM in distinct ways.

Post-hoc tests of the Chi-square test revealed significant differences in applying the HBM constructs across sources of credibility (χ^2 ^= 39.947, **p* *< 0.001), as detailed in Table 6. Letter-based multiple comparisons revealed that pregnant and postpartum women used self-efficacy strategies in posts significantly more frequently than fitness professionals and commercial entities (23.1% vs. 9.6% vs. 8.3%, respectively; *p* < 0.05). In addition, commercial entities referenced exercise-related barriers more frequently than pregnant and postpartum women (36.7% vs. 24.1%, respectively; *p* < 0.05).

**Table 6:** Usage of HBM framework by different information sources.

		Healthcare Professionals	Pregnant and Postpartum Women	Fitness Professionals	Ordinary Individuals	Commercial Entities
**HBM**	Severity	5(5.4%)^a^	68(8.5%)^a^	13(13.8%)^a^	2(4.5%)^a^	8(7.3%)^a^
	Susceptibility	3(3.3%)^a^	32(4.0%)^a^	9(9.6%)^a^	1(2.3%)^a^	6(5.5%)^a^
	Benefits	38(41.3%)^a^	321(40.3%)^a^	38(40.4%)^a^	17(38.6%)^a^	46(42.2%)^a^
	Barriers	29(31.5%)^ab^	192(24.1%)^b^	25(26.6%)^ab^	18(40.9%)^ab^	40(36.7%)^a^
	Self-Efficacy	17(18.5%)^ab^	184(23.1%)^b^	9(9.6%)^a^	6(13.6%)^ab^	9(8.3%)^a^

Since five cells (20.0%) had expected counts of less than 5, Fisher’s exact test yielded χ² = 39.947, **p* *< 0.001. In each row, different letters indicate significant differences between these categories.

Given that the sources of information are heuristic cues in the HSM framework, only the differences between the sources of information in terms of systematic cues were tested. As shown in Table 7, there was also a significant difference in the quality of information among different sources (χ^2 ^= 11.846, *p = *0.023). According to letter-based multiple comparisons, posts by pregnant and postpartum women exhibited significantly higher information completeness (77.3% vs. 55.8%) but lower information accuracy (22.7% vs. 44.2%) compared to those by fitness professionals (*p* < 0.05).

**Table 7:** Information quality of HSM by different information sources.

		Healthcare Professionals	Pregnant and Postpartum Women	Fitness Professionals	Ordinary Individuals	Commercial Entities
Information Quality	Completeness	35(79.5%)^ab^	256(77.3%)^b^	24(55.8%)^a^	18(75%)^ab^	35(66%)^ab^
	Accuracy	9(20.5%)^ab^	75(22.7%)^b^	19(44.2%)^a^	6(25%)^ab^	18(34%)^ab^

Since five cells (20.0%) had expected counts of less than 5, Fisher’s exact test yielded χ² = 11.846, p = 0.023. In each row, different letters indicate significant differences between these categories.

## 5 Discussion

This study aims to investigate the extent to which HBM and HSM strategies are used to promote prenatal exercise on social media platforms and analyze their impact on social media public engagement. Results showed that most of the HBM constructs and some of the HSM strategies effectively promoted prenatal exercise on social media.

### 5.1 Impact of HBM and HSM construct application

First, unlike previous studies on health communication, the information on exercise during pregnancy on social media seems to rarely emphasize the severity and susceptibility of adverse consequences caused by lack of prenatal exercise. Compared to posts that did not emphasize them, posts that emphasized the severity and susceptibility did not significantly attract public engagement. Considering the particularity of pregnancy, one possibility is that emphasizing the severity and susceptibility of adverse consequences caused by lack of exercise during pregnancy may exacerbate women’s anxiety about the pregnancy process, thereby triggering their avoidance mechanisms.

Second, benefits, barriers, and self-efficacy were the three most frequently mentioned constructs in social media messages about prenatal exercise. Moreover, messages that included these constructs received significantly more public engagement than those that did not. Specifically, cues to benefits and self-efficacy positively influenced public engagement, whereas barriers negatively influenced public engagement, which is consistent with past research [31].

Third, fitness professionals and commercial entities with relatively low credibility were less likely to use self-efficacy strategies in their posts than pregnant women with high credibility. This may be because pregnant women prefer to share their personal experiences and practical skills, and their content is more narrative and emotionally resonant. For example, “I have improved my body through exercise, and so can you!” However, the purpose of fitness professionals and commercial entities is commercial, so they may focus more on showcasing the effects of exercise to attract users rather than helping users overcome psychological barriers and build exercise confidence. In addition, commercial entities tended to refer more often to barriers to exercise. This may be because mentioning exercise barriers can lead to corresponding solutions, such as launching professional pregnancy exercise courses and offering one-on-one pregnancy exercise guidance services.

Fourth, the current study reveals two alarming phenomena: Firstly, the findings show that healthcare professionals, the most trusted sources, are infrequent in disseminating information about exercise during pregnancy on social media. Previous research has pointed out that the lack of support and information from healthcare professionals is partly responsible for pregnant women’s inadequate exercise during pregnancy [56]. Additionally, most posts in our study are posted by pregnant and postpartum women, who have posted a certain amount of inaccurate information. However, when this inaccurate information originates from sources trusted by the audience, i.e., pregnant and postpartum women, the negative effects will be far-reaching. They may be more likely to mislead the audience’s perceptions and judgments about exercise during pregnancy than messages from distrusted sources. Due to the potential for this inaccurate information to lead to unhealthy behaviors among people [57], we should give them the utmost attention.

### 5.2 Theoretical and practical contributions

This study has both theoretical and practical significance for disseminating and popularizing pregnancy exercise information on social media.

First, this study expands the application fields of the Health Belief Model (HBM) and Health Behavior Change Model (HSM) to prenatal exercise promotion. To the best of our knowledge, no systematic analysis of the persuasive strategies and dissemination effects of pregnancy exercise information has been conducted online. Additionally, few studies have explored the potential impact of social media exposure on pregnancy exercise behavior. This study partially fills the gap in this research direction and provides a theoretical basis for developing and optimizing social media dissemination strategies for pregnancy exercise information.

Second, this study also has some practical implications. Initially, it is important to pay attention to strategies in message design, such as minimizing the impact of perceived barriers and increasing audience confidence and beliefs by emphasizing self-efficacy and the benefits of exercise during pregnancy. However, it is worth noting that while emphasizing the benefits to pregnant women’s appearance and body image in the short term appears to significantly enhance communication effectiveness, this strategy also indirectly reflects the potential impact of social norms and pressures on them.

Specifically, it may lead pregnant women to overly focus on their appearance and body shape changes, which may induce varying degrees of appearance anxiety and may even undermine the importance they place on their physical and mental health. Therefore, this phenomenon should be a concern in our subsequent research and practice interventions. Subsequently, attention should be paid to the quality of the information when designing the message to provide pregnant women with as much accurate and sufficient information about exercise during pregnancy as possible [25]. Although the accuracy of information may not directly and significantly affect immediate public engagement, it nevertheless poses a potential threat to the normative and scientific nature of pregnancy exercise promotion information, which may undermine the overall effectiveness of this type of health communication.

Additionally, it is necessary to fully leverage heuristic cues, such as the source’s credibility, in disseminating prenatal exercise information on social media. On the one hand, it is necessary to provide systematic training for healthcare professionals, update their knowledge promptly, and ensure that they can provide accurate prenatal exercise guidance. At the same time, conditions should be created to incentivize healthcare professionals to use social media platforms to publish popular scientific information related to prenatal exercise.

### 5.3 Study limitations and future research directions

This study has the following limitations that could be addressed by future research.

First, this study used likes and favorites as metrics for public engagement. These indicators may be potentially influenced by algorithms on the Xiaohongshu platform (such as prioritizing highly interactive content) and user preferences (such as favoring positive and practical information), leading to underestimation of content that emphasizes health threats. Future research can combine algorithm mechanisms and use interviews to more comprehensively evaluate communication effectiveness. Future research could also examine how social media platforms, channels, and cultural contexts influence the effectiveness of prenatal exercise information dissemination.

Second, this study did not control for the total number of posts per account because the platforms did not provide such data. Considering that accounts with a high posting frequency may reach a larger audience, future research on other platforms may include the total number of posts as a control variable to improve analytical rigor.

Third, although we found an important fact that healthcare professionals had low engagement on this topic on social media, we did not explore the reasons behind it. Future research can examine the barriers hindering healthcare professionals from participating in social media platforms like Xiaohongshu.

## 6 Conclusion

Our study found that HBM factors such as self-efficacy, benefits, and barriers significantly impact audience engagement in pregnancy exercise promotion information on social media. Pregnant and postpartum women are the main sources of information on pregnancy exercises on social media, while information posted by healthcare professionals is most likely to attract audience participation. In addition, this study also found room for improvement in the accuracy of the information about exercise during pregnancy. Therefore, when designing information related to prenatal exercise, information publishers should consider the accuracy of the information so that high-quality information can reach the audience, thereby improving public awareness of prenatal exercise, enhancing social recognition and support, and ultimately improving the rate of prenatal exercise, contributing to women's health and well-being.

## Supporting information

S1 Data Minimal anonymized dataset for study replication. (XLSX)
